# 4D-Printed Smart Materials in Clear Aligner Fabrication: A Comprehensive Review -

**DOI:** 10.31661/gmj.v14iSP1.3918

**Published:** 2025-12-15

**Authors:** Hossein Ebrahimi, Setareh Khosravi

**Affiliations:** ^1^ Department of Orthodontics, School of Dentistry, Shahed University of Medical Sciences, Tehran, Iran; ^2^ Department of Orthodontics, School of Dentistry, Alborz University of Medical Sciences, Karaj, Iran

**Keywords:** Biomaterials, Clear Aligners, Digital Orthodontics, Shape-memory, Smart Material, 3D-printing

## Abstract

Contemporary dentistry is shifting from passive materials toward biocompatible alternatives with superior mechanical and chemical properties. Smart materials, capable of responding to external stimuli such as temperature, pH, moisture, light, mechanical stress, and electromagnetic or biological signals, are at the forefront of this evolution. Their integration into additive manufacturing has given rise to "4D printing," where printed structures can change over time in response to environmental conditions. In orthodontics, this innovation enables the direct 3D printing of clear aligners, offering precise control over thickness, fit, and design while eliminating thermoforming steps. This results in greater geometric accuracy and workflow efficiency. This review aims to highlight the emerging role of smart materials in clear aligner therapy, focusing on their clinical potential and future applications within the evolving landscape of digital orthodontic.

## Introduction

In the past, dental materials were mainly chosen for their chemical stability and lack of interaction with oral tissues. Nowadays, the focus has shifted toward biocompatible options that combine safety with enhanced mechanical and chemical performance [1][2]. Clear aligner fabrication is the process of making transparent orthodontic appliances to align teeth [3]. Biocompatible materials are generally grouped into bioinert, bioactive, and smart (bioresponsive) types based on how they interact with the biological environment [1][2][3]. In recent years, growing interest has emerged in replacing passive dental materials with smart alternatives capable of responding to external stimuli by altering their shape, color, or size [4][5]. Smart materials are capable of altering one or more of their properties in response to external stimuli such as energy absorption or environmental changes, which classifies them as responsive materials [6][7]. In the dynamic oral environment with fluctuations in pH, humidity, and microbial activity, there is increasing demand for such materials that can adapt beneficially to these variations [8]. Their application has notably transformed orthodontics, particularly through the use of shape memory alloys and polymers [9][10]. 4D printing involves three spatial dimensions plus time, allowing objects to change shape or function in response to stimuli, unlike 3D printing, which creates static objects with only three spatial dimensions. Smart materials play a key role in 4D printing, in which structures get created that respond to changing conditions. However, in dentistry and medicine, this technology is still under development [11]. Although direct 3D printing of clear aligners is an emerging field, with limited clinical studies available, challenges related to surface roughness and material properties remain, as experimental studies consistently show that the surface quality of printed aligners often fails to meet clinical standards without optimized post-processing [12]. In this review, we attempted to reveal the possibility of using smart materials in the field of orthodontic clear aligner treatments as a beneficial material for use in 3D or 4D printing approaches.

## Clear Aligner Materials and 3D-printing

**Table:** Table1. Common Smart Polymers in 3D printing Clear Aligners.

**Brands**	**Manufacturer**	**Composition**
**E-Guard**	Envision TEC (Rockhill, SC, United States)	Photo-polymeric clear methacrylate-based resin
**Dental LT**	Form Labs (Somerville, Massachusetts)	Photopolymers methacrylate-based resin
**TC-85A** **(Tera Harz TC-85) **	Graphy (Seoul, South Korea)	Aliphatic vinyl ester-polyurethane polymer
**3D:1M **	Okamoto chemicals	Aliphatic vinyl ester-polyurethane polymer
**Accura 60 SLA**	3D systems (Rockhill, South Carolina)	Polycarbonate-based photopolymer

**Table:** Table2. Comparison of the advantages and disadvantages of thermoplastic and directly 3D-printed clear aligner materials [102][103]

**3D Printed Materials**		**Thermoplastic Materials **
**Advantages:** - High production efficiency - Customizable thickness according to requirements - Environmentally friendly manufacturing process - Reduction in material waste **Disadvantages:** - Requires post-curing processing - Limited availability of dedicated design software - Not widely available commercially - Materials have not undergone sufficient clinical trials - Inaccurate printing direction may affect outcomes - Final thickness greater than the designed specifications		**Advantages:** - Good biocompatibility - Wide range of approved materials - Accessibility **Disadvantages:** - Generation of waste materials - Potential environmental pollution - The final product has thinner dimensions than originally designed - Long production process - Alterations in material properties during processing

Clear aligners can be fabricated using two primary approaches: the conventional technique, which utilizes vacuum thermoforming of thermoplastic sheets over either 3D-printed or cast dental models, or the direct 3D-printing method, which bypasses the need for physical intermediary models altogether [13][14]. The materials used in clear aligner thermoforming fabrication have evolved from "polyurethane" and "polyethylene terephthalate glycol-modified (PETG)" to materials like "polypropylene (PP)," "polycarbonate (PC)," and "thermoplastic polyurethanes (TPU)"[15]. However, none of these materials possess all the ideal characteristics, indicating the need for a new material to optimize orthodontic treatment [16]. Thermoforming variations affect thermoplastic properties, impacting aligner fit and performance [17]. The process also raises environmental concerns like plastic waste, high energy use, and toxic emissions (benzene from PETG, tetrahydrofuran from polyurethane) [18][19]. Mechanical friction may release microplastics, but current evidence suggests this is minimal during the short wear time of aligners [20]. To address the drawbacks of traditional vacuum thermoforming, direct 3D printing of clear aligners has emerged as a promising alternative [21]. This approach eliminates mechanical distortions and material degradation caused by thermoforming, resulting in superior dimensional accuracy, better fit, increased mechanical strength, and enhanced reproducibility [22]. Consequently, digital technologies such as 3D printing have become favored methods for producing dental aligners when applicable that offers more precision, customization, and manufacturing efficiency [23]. Direct 3D printing circumvents the negative effects associated with thermoforming, including changes in mechanical, dimensional, and aesthetic properties, thereby providing improved geometric accuracy, fit, efficacy, and consistency. Common materials used in orthodontic 3D printing include acrylonitrile-butadiene-styrene (ABS), epoxy-based stereolithography resins, polylactic acid (PLA), polyamide (nylon), glass-filled polyamide, as well as metals like silver, steel, titanium, photopolymers, wax, and polycarbonates [24][25][26].

Additive Manufacturing (AM) refers to technologies that create objects directly from digital models, beginning with CAD files converted into STL format to guide the printing process [26]. AM, also referred to as 3D printing, rapid prototyping, or direct digital manufacturing, provides significant advantages by enabling the fabrication of complex geometries and highly customized components tailored to specific applications or patients [27][28]. The advent of smart materials has enabled AM to evolve into "4D printing," which incorporates structural transformation over time [29][30]. Unlike traditional 3D printing, 4D printing allows fabricated objects to change shape or function in response to external stimuli such as temperature, light, moisture, pH, or electromagnetic fields [31][32][33]. These changes can be pre-programmed and precisely controlled that gives material capability of the development of getting into adaptable structures with dynamic geometries and time-responsive functionality [34].

**Figure-1:**
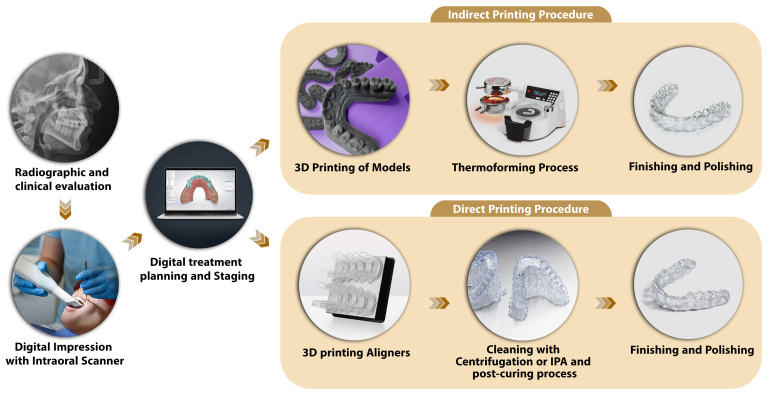
Figure 1. Step-by-Step Workflow of Indirect vs. Direct 3D Printing Process Used in Orthodontic Clear Aligner Fabrication

The 3D printing (Direct Printing) workflow for clear aligners using the direct printing approach includes five main steps: Acquisition of Digital Files (Digital impressions are obtained via intraoral scanning technologies following clinical and radiographic evaluations), Digital Treatment Planning (Treatment objectives are defined, and necessary components, such as attachments, are digitally designed using specialized software), 3D Printing of Clear Aligners (The digitally designed models are fabricated through 3D printing using appropriate resins and printer technologies), Post-Curing Processing (Printed models are detached from the build platform, and any excess structures are removed. At this stage, residual uncured resin is eliminated using isopropyl alcohol (IPA) or centrifugal methods), Finishing and Polishing (Sharp or rough edges are refined to ensure proper fit, patient comfort, and aesthetic quality of the final appliance) [35]. Direct 3D printing of clear aligners allows precise in-house fabrication that improves accuracy and efficiency by removing thermoforming steps [36][37]. Combining this with 4D printing and smart materials enables adaptive, programmable orthodontic devices [38]. Figure-1 presents a comparative workflow between the thermoforming process (indirect printing) and direct 3D printing. Table-1 summarizes the commonly used polymers for 3D-printed clear aligners, while Table-2 compares the advantages and disadvantages of thermoplastic versus directly 3D-printed clear aligner materials.

## Smart Materials

**Table:** Table3. Desirable properties and characteristics of a material for clear aligner fabrication.

**Category**	**Property/Characteristic**	**Clinical Relevance**
	- Optimal stiffness and elasticity	Sufficient force for tooth movement without compromising comfort
	- High resilience and flexibility	Maintains shape while adapting to tooth morphology
**Mechanical**	- Adequate stress and distortion resistance	Withstands chewing and occlusal forces without permanent deformation
	- Sustained force delivery	Delivers continuous, gentle force for effective tooth movement
	- Optimal Fitting And Accuracy	Provides a precise fit for accurate and predictable tooth movement
	- Dimensional stability	Retains form during treatment duration and under thermal stress
**Structrual**	- Appropriate aligner thickness	Provides effective force while maintaining comfort and aesthetics
	- Stain and color resistance	Resists stains and maintains high transparency for aesthetic appearance
	- High transparency	Aesthetically acceptable and less visible during wear
	- Thermal resistance	Withstands heat from fabrication and oral temperature variations
	- Resistance to oral chemicals, enzymes, and beverages.	Prevents degradation and maintains integrity in the oral environment
**Chemical**	- Low cytotoxicity and high biocompatibility	Ensures safe intraoral use and minimizes adverse tissue responses
	- Antimicrobial Properties	Inhibits microbial growth, enhancing hygiene and aligner material longevity.

The clinical success of clear aligners heavily depends on the physical and mechanical properties of the materials used in their fabrication [39]. An ideal aligner material should exhibit a balance of resilience, elasticity, biocompatibility, and transparency while withstanding mechanical, thermal, and chemical stresses. Additionally, it must possess sufficient stiffness to deliver the forces required for effective tooth movement. Materials with excessively high modulus of elasticity may result in rigid, inflexible aligners, whereas insufficient stiffness leads to inadequate force generation for tooth repositioning [40]. Table-3 outlines the essential characteristics of an optimal aligner material.

Improvements in the biochemical composition of aligner materials can significantly enhance their therapeutic effectiveness; without such advancements, aligners remain constrained by biomechanical limitations and may underperform compared to fixed orthodontic appliances [41]. The next section explores the primary factors that affect the critical properties of smart materials, a key step toward optimizing their design.

### Printing Process, Post-printing Process, 3D Printer Machine

The mechanical properties of 3D-printed materials are influenced by the printing technique, post-processing protocols, and printer type, potentially impacting clinical outcomes [42][43][44]. Aligners fabricated directly from Stereolithography (STL) files have demonstrated superior accuracy and precision compared to those produced via vacuum thermoforming [25]. While some studies report that print orientation has minimal impact on mechanical properties such as flexural strength [45][46] and only a slight effect on overall accuracy [47], another study has found that it significantly influences the dimensional accuracy of directly printed orthodontic aligners [48]. Horizontal print orientation, in particular, is associated with optimal mechanical performance [49]. Moreover, the printing angle affects both resin consumption and production costs [50]. A study by Mattle et al. found that the mechanical properties of 3D-printed resin aligners were not significantly affected by either post-curing in a nitrogen atmosphere or by heat treatment [51]. Similarly, one study reported that eliminating oxygen during the printing process did not influence the mechanical properties of the aligners [52]. However, another study has reported conflicting results regarding the impact of oxygen exposure during printing, leaving this issue open to further investigation [53]. Research has also demonstrated that optimal polymerization times significantly affect both the mechanical and thermal properties of dental resins, with materials like Tera Harz benefiting from both very short and extended curing durations [54]. Regarding post-processing, a 2-minute centrifugation at 55°C has been proposed as an effective method to remove uncured resin without adversely affecting the aligners’ physical or optical qualities, making it practical for clinical applications [55][56]. A minimum post-curing time of 60 minutes is essential to enhance the clinical performance of 3D-printed resins [57]. Ultraviolet post-curing is also critical for achieving the necessary rigidity in directly printed aligners, though prolonged curing has limited impact on accuracy [58]. Furthermore, the type of 3D printer plays a crucial role in determining the precision of orthodontic models and the mechanical properties of printed aligners, which in turn impact clinical outcomes and treatment effectiveness [42][43]. On the other hand, 3D printing resins are highly toxic before polymerization, but their toxicity decreases after curing, making proper post-processing essential to reduce toxicity to safe levels [22]. An in-vivo studies confirm the general safety of several 3D-printed materials [44], though material choice and post-processing significantly affect in vitro cytotoxicity [59]. Another trial found no cytotoxic differences using various UV-polymerization units or rinsing solvents [60]. Prolonged UV exposure and extended curing increase cytotoxicity which might be showing the need for standardized curing protocols [61]; for example, a 20-minute UV cure ensures safety for Tera Harz TA-28 up to 6 mm thick [62]. Among common polymers, TC-85A showed no cytotoxicity, while E-Guard and Dental LT exhibited slight effects [63][64].

### Material Composition

The introduction of 3D-printed aligners with shape memory properties (4D aligners) marks a significant advancement in orthodontics. These materials demonstrate mechanical characteristics better suited for orthodontic applications compared to traditional thermoforming materials [65]. The composition of aligner materials plays a crucial role in determining the forces and moments they can apply [66]. For instance, Dental LT resin, when adequately cured, produces geometrically precise aligners with improved mechanical strength and elasticity that enables efficient and accurate in-house fabrication [36]. TC-85 3D-printed aligners have proven effective in delivering forces appropriate for tooth movement [67][68]. This advanced material exhibits exceptional shape memory at oral temperatures, enhancing aligner fit, minimizing force decay, and permitting greater tooth movement per step thanks to its increased flexibility. Moreover, TC-85 maintains microhardness comparable to conventional thermoformed sheets, ensuring durability and clinical effectiveness over time [67]. Its wider elastic range and enhanced flexibility also allow for more extensive tooth movements without causing permanent deformation [13].

### Viscosity

The quality of 3D prints, including accuracy, durability, and aesthetic appeal, depends significantly on the viscosity of the resin used [68]. The 3D printing resin showed a significant decrease in viscosity and a 2.34-fold increase in flow when heated to 55 °C. This behavior, consistent with typical liquid resins, highlights a direct relationship between temperature, viscosity, and flow. When combined with shear force, the resin’s viscosity approached zero, suggesting that raising the temperature can enhance the efficiency of centrifugal cleaning systems [55]. Increasing the oligomer content in the resin system effectively improves mechanical properties; however, it also significantly raises the viscosity of the UV-curable resin, which negatively impacts its printability [69]. IV. Aligner Thickness Shape-memory 3D-printed materials (4D aligners) have introduced a transformative shift in aligner fabrication, offering superior mechanical properties, optimized workflow, and improved control over thickness and design compared to conventional thermoforming techniques [67]. Direct 3D printing offers enhanced precision by eliminating intermediate steps, enabling full control over aligner thickness, coverage, and attachment placement [70]. With 0.4 mm-thick aligners delivering forces between 3.1 N and 15.8 N, and thicker aligners producing higher forces [66]. While increasing aligner thickness can affect force and movement generation, the relationship is complex and tooth-specific [55][71][72]. Multilayer materials tend to produce lower initial forces than single-layer ones [73], and increased thickness (e.g., 0.7 mm vs. 0.5 mm) correlates with improved bending resistance [45]. Although some researchers suggest that thickness has minimal influence under specific settings [74]; others report significant impacts on mechanical behavior, color stability, and surface roughness [75]. Additionally, thermoforming typically reduces the original material thickness, whereas direct 3D printing may inadvertently increase thickness, potentially compromising clinical performance [76][77]. Increased thickness also improves retention, making thicker, and single-layer rigid materials preferable for effective bodily tooth movement [78]. Thicker aligners produce greater forces and possess a higher modulus of elasticity with reduced deformation, making them more suitable for complex tooth movements such as root translation. Conversely, thinner aligners offer increased flexibility and deformability but are more prone to fracture. Notably, in 3D-printed aligners, reducing the printing layer thickness enhances strength that shows the critical influence of thickness on force generation and material performance [68]. Direct-printed aligners provide biologically compatible and more consistent forces for tooth movement compared to thermoformed ones under in vitro conditions [79].

Thermomechanical aging reduces these forces within the first 48 hours [73][80]. Moreover, direct 3D printing allows precise customization of thickness and addition of ridges, enhancing biomechanical efficiency and potentially reducing the need for attachments [81].

### Aging

Orthodontists should consider that aligner materials may deteriorate over time, affecting mechanical performance and guiding optimal replacement intervals [73]. However, one study reported no significant mechanical changes after one week of intraoral use of in-house 3D-printed aligners [82]. A 15-day wear period is recommended for a better fit and minimal gaps [83]. Thermoforming and aging both influence the mechanical properties of aligner materials, with thermoforming having a more pronounced weakening effect [73]. Despite this, thermoformed aligners have demonstrated good thickness retention and dimensional stability following intraoral aging in healthy adult subjects [84]. Nonetheless, biocompatible 3D-printed resins maintain sufficient strength to endure occlusal forces even after aging, making them suitable for intraoral appliances [85].

### Water Absorption

Moisture, specifically a simulated oral conditions, has a more pronounced impact on the mechanical properties of direct 3D-printed aligners than on thermoformed ones, potentially compromising their ability to deliver consistent orthodontic forces [86]. In contrast, thermoplastic materials generally demonstrate lower water absorption and solubility, along with smoother surfaces, resulting in improved transparency and color stability compared to evaluated 3D-printing resins [87].

### Surface Patterns and Abrasion

Aligner materials must resist degradation in the oral environment to withstand the forces generated during chewing [88]. A research has shown that intraoral exposure and functional use can significantly impact the surface roughness of "in-house" fabricated aligners across different regions [89]. Although minor surface defects often appear after two weeks of clinical use, these changes do not seem to markedly affect the mechanical properties of the aligners [90]. The surface characteristics of clear aligners are influenced by both manufacturing techniques and process parameters. Thermoformed and 3D-printed aligners differ notably in thickness and fit depending on tooth type and location, with thermoformed materials generally being stiffer, harder, and sometimes rougher [14][91]. However, surface texture alone does not appear to significantly influence the force delivery characteristics in either thermoformed or directly printed aligners [79]. Recent research by Goracci et al. highlights the importance of print orientation on surface roughness and gloss, with vertical printing yielding significantly rougher and glossier surfaces compared to horizontal printing. The material type mainly affects gloss, with TC material showing higher gloss than LT. Moreover, polishing enhances the specimens’ resistance to aging, which may contribute to improved clinical longevity [92][93].

### Discoloration

Orthodontic aligners are prone to staining from beverages like coffee, cola, and red wine [94][95]. Patients should limit such intake during treatment [96]. Optical properties vary by brand and material, and degrade with aging [97]. The optical properties of orthodontic aligners vary across different brands and materials but tend to degrade with in vitro aging. Studies indicate that a 30-minute post-curing period can achieve clinically acceptable color stability, highlighting the need to optimize post-curing protocols according to clinical requirements. Additionally, increased material thickness has been linked to greater yellowing in the samples [98]. Comparative studies show that 3D-printed aligners, especially those made from polyurethane, undergo more discoloration than thermoformed ones, while PETG-coated aligners demonstrate greater resistance to staining and degradation [99].

### Permanent Deformations

An ideal orthodontic aligner should exhibit sufficient rigidity, high yield strength, and deliver forces within the elastic range. Common aligner materials, however, have an elastic modulus 40-50 times lower than Ni-Tiarchwires, making them more prone to permanent deformation [100]. Force decay in clear aligners arises from viscoelastic behavior and repeated use. TC-85 aligners, with shape memory properties, maintain consistent force at body temperature and support greater tooth movement due to their superior flexibility and wider elastic range [13][101].

## Conclusion

The integration of smart materials, particularly shape memory polymers like TC-85, introduces a promising paradigm shift by enabling clear aligners to actively respond to the oral environment. These materials exhibit improved elasticity, force consistency, and shape retention, which could allow for fewer aligner stages and better patient outcomes. Factors such as material composition, thickness, aging resistance, and water absorption critically influence performance and must be carefully considered during material selection and processing. Despite the promising advances, the clinical use of smart materials in clear aligners remains in its early stages. More in vivo studies and long-term evaluations are required to validate their effectiveness, biocompatibility, and safety. Future research should focus on standardizing printing protocols, enhancing material transparency and stain resistance, and developing environmentally friendly formulations. Ultimately, the convergence of digital workflows, additive manufacturing, and smart materials can revolutionize orthodontic care, making treatment more efficient, personalized, and biologically harmonious.

## Conflict of Interest

The authors of this manuscript declare that they have no conflicts of interest, real or perceived, financial or nonfinancial in this article.
